# Resected thymic large cell neuroendocrine carcinoma: report of a case

**DOI:** 10.1186/s40792-018-0540-2

**Published:** 2018-11-16

**Authors:** Hiromitsu Domen, Yasuhiro Hida, Masaaki Sato, Haruka Takahashi, Tatsuru Ishikawa, Yosuke Shionoya, Midori Hashimoto, Kaoru Nishiyama, Yuma Aoki, Kazuho Inoko, Syotaro Furukawa, Kazuomi Ichinokawa, Hidehisa Yamada

**Affiliations:** 1 Department of Surgery, Nippon Telegraph and Telephone East Corporation Sapporo Medical Center, South 1, West 15, Chuo-ku, Sapporo, Hokkaido 060-0061 Japan; 2 Department of Cardiovascular and Thoracic Surgery, Faculty of Medicine, Hokkaido University, North 14, West 5, Kita-ku, Sapporo, Hokkaido 060-8648 Japan; 3 Department of Pathology, Nippon Telegraph and Telephone East Corporation Sapporo Medical Center, South 1, West 15, Chuo-ku, Sapporo, Hokkaido 060-0061 Japan; 4 Department of Respiratory Medicine, Nippon Telegraph and Telephone East Corporation Sapporo Medical Center, South 1, West 15, Chuo-ku, Sapporo, Hokkaido 060-0061 Japan

**Keywords:** Large cell neuroendocrine carcinoma, Thymus, Surgery, LCNEC, Thymic LCNEC

## Abstract

**Background:**

Thymic large cell neuroendocrine carcinoma (LCNEC) is extremely rare. The detailed clinical features of thymic LNCECs remain unknown.

**Case presentation:**

A 90-year-old man with a history of diabetes mellitus, chronic renal failure, and an abdominal aortic aneurysm underwent computed tomography for follow-up, which showed an anterior mediastinal tumor, measuring 31 mm × 28 mm in diameter. Magnetic resonance imaging showed an iso-intensity mass on T1-weighted images and high intensity on T2-weighted images. 18F-Fluorodeoxyglucose-positron emission tomography showed marked uptake in the mass, which was diagnosed as invasive thymoma or thymic carcinoma. Video-assisted thoracic surgery through the left thoracic cavity was converted to median sternotomy due to severe adhesions between the left lung and the chest wall. Partial thymectomy and combined partial resection of left upper lobectomy and the first and the second costal cartilages were performed. The pathologic diagnosis was thymic LCNEC, Masaoka stage III. The patient developed pleural dissemination and left lung metastases in 5 months and died 12 months after surgery.

**Conclusions:**

Thymic LCNEC has high malignant potential. More cases need to be studied.

## Background

Primary thymic neuroendocrine carcinomas (NECs) were categorized under the rubric of “thymomas” until 1972 when Rosai and Higa suggested that these tumors were sufficiently distinctive to warrant classification as carcinoid tumors [1]. In 1999, the World Health Organization established thymic epithelial tumor criteria and reclassified thymic carcinoma, referring to NECs as a subtype [2]. In particular, the large cell neuroendocrine carcinoma (LCNEC) was subclassified in the thymic NECs in accordance with the classification of pulmonary NECs. Detailed clinical features of thymic LNCECs remain unknown, because of their extreme rarity. A surgical case is presented along with a review of the literature.

## Case presentation

**Fig. 1:**
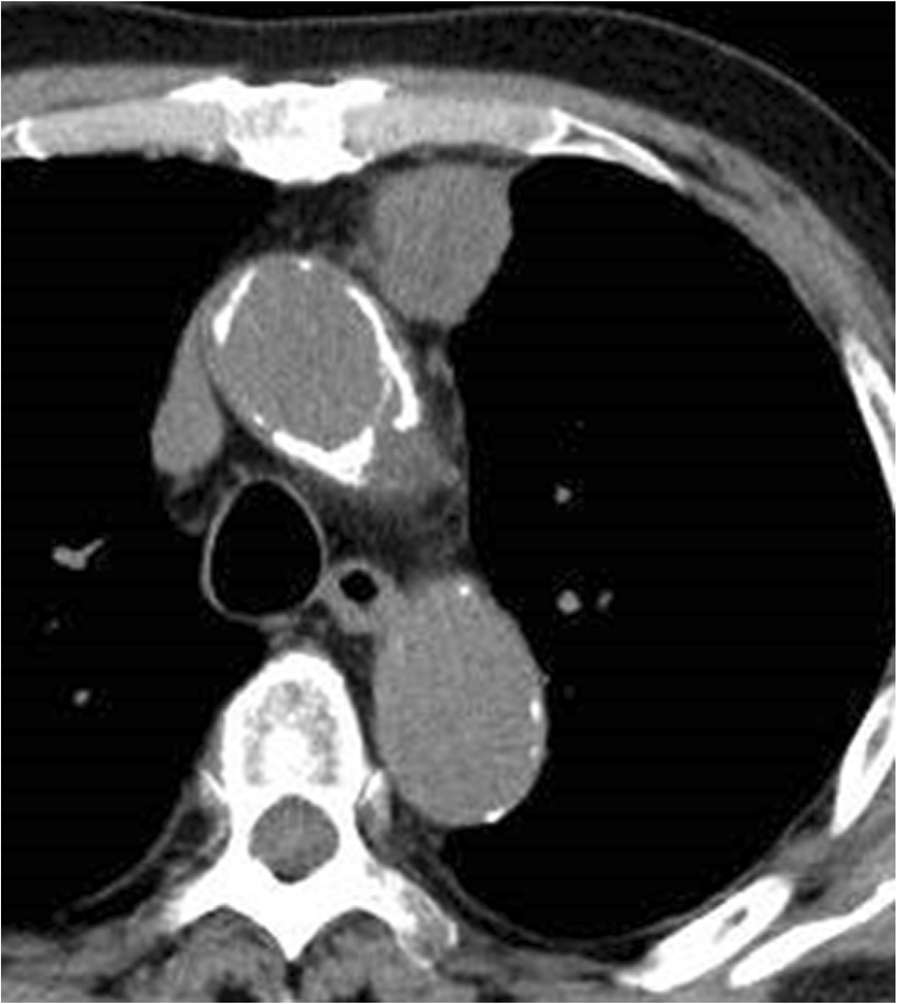
CT findings. CT shows a 31 mm × 28 mm, rough-shaped mass in the anterior mediastinum. It is located mainly in the mediastinum, not in the lung

**Fig. 2:**
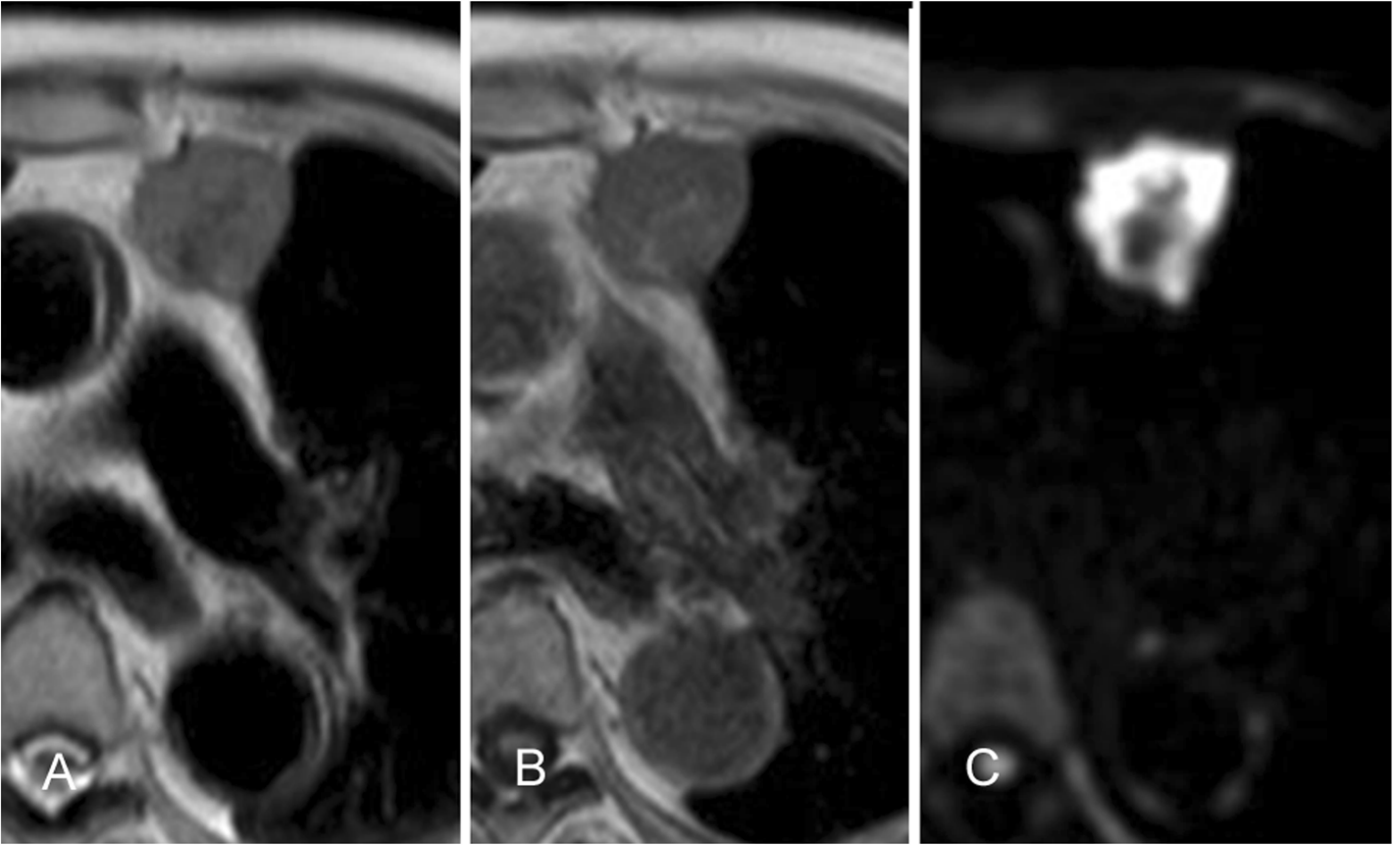
MRI findings. MRI shows the iso-intensity mass on T1-weighted imaging (**a**) and high intensity on T2-weighted imaging (**b**). The marginal zone of the mass shows a low-density area with a refined lobulated structure. Diffusion-weighted image shows a high intensity area in the marginal zone with apparent diffusion coefficient sequences (C)

**Fig. 3:**
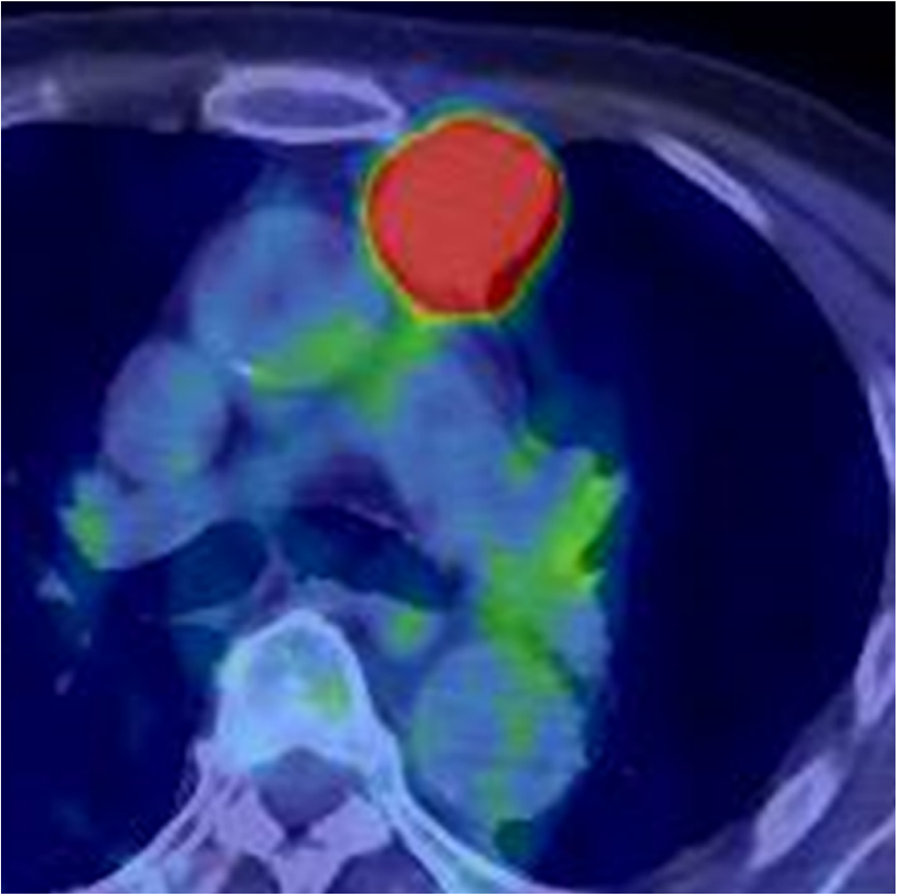
FDG-PET findings. FDG-PET shows that the mass has a high FDG uptake value (SUVmax 30.5). No organs have a high uptake value

**Fig. 4:**
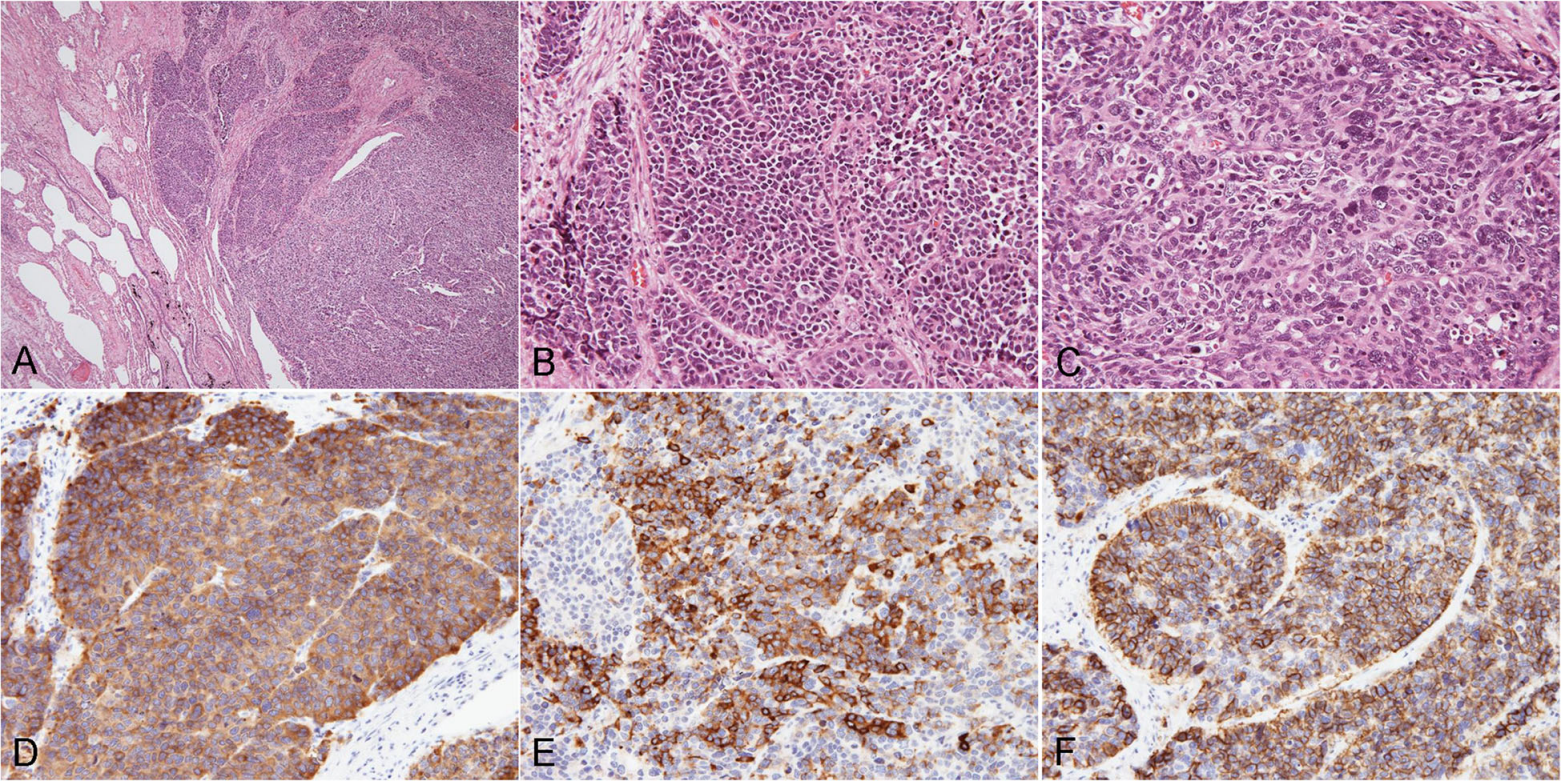
Histopathologic findings. Atypical cell nests with large nuclei are seen with lung invasion (**a**). Tumor cells form nests showing a palisading or organoid pattern (**b**). Cells with bizarre or multiple nuclei are also seen (**c**). Immunohistochemistry shows positive staining for CD56 (**d**), chromogranin A (**e**), synaptophysin (**f**), and TTF-1 and negative staining for CD5 and p40. The pathologic diagnosis is large cell neuroendocrine carcinoma of the thymus

A 90-year-old man had been followed by a cardiologist because of diabetes mellitus, chronic renal failure, and an abdominal aortic aneurysm. A solid mass was found on plain computed tomography (CT) at a regular health check-up. He had smoked 20 cigarettes per day for 45 years. Plain CT showed a solid mass, 31 mm × 28 mm, with a partially unclear margin with the normal thymic tissue in the anterior mediastinum (Fig. 1). Magnetic resonance imaging (MRI) showed an iso-intensity mass on T1-weighted images and high intensity on T2-weighted images (Fig. 2). Diffusion-weighted imaging showed a high-intensity area in the marginal zone, with apparent diffusion coefficient sequences. Laboratory findings and results for markers such as alpha-fetoprotein, beta-human chorionic gonadotropin, anti-acetylcholine receptor antibody, and soluble interleukin-2 receptor were not significant preoperatively. 18F-Fluorodeoxyglucose-positron emission tomography (FDG-PET) showed the mass had marked uptake of FDG, early maximum standardized uptake value (SUVmax) of the mass 30.5 (Fig. 3). The mass was thought most likely to represent thymic cancer, followed by invasive thymoma, Masaoka stage II, and UICC-T1bN0M0 stage I. First, video-assisted thoracic surgery (VATS) was tried through the left pleural cavity. Strong and broad adhesions between the left lung and the chest wall were observed. Since VATS appeared risky, the procedure was converted to median sternotomy. An anterior mediastinal tumor was fixed to the anterior chest wall. We attempted dissection in the extrapleural layer, but the tissue was not easily dissected. The tumor seemed to be invading into the left upper lobe of the lung and the chest wall. We abandoned dissection at once. Partial thymectomy, with combined partial resection involving left upper lobectomy and the first and the second costal cartilages, was done. Operation time was 4 h and 29 min, and blood loss volume was 450 ml. The patient’s postoperative course was uneventful. Histopathologic examination showed a white, solid, 35 × 30 × 25 mm^3^ mass with regional bleeding and necrosis (Fig. 4). Microscopically, the tumor nests composed of atypical cells with large nuclei showed a palisading or organoid pattern. Cells with bizarre or multiple nuclei were also seen. Forty-fifth mitoses per 2 mm^2^ and broad necrosis were seen. The surgical margin was free from tumor cells. Immunohistochemistry showed positive staining for chromogranin A, synaptophysin, and CD56 and negative staining for CD5 and p40. The tumor cells also showed positive nuclear staining for thyroid transcription factor-1 (TTF-1). Histology proved the tumor invasion to the left upper lobe of the lung but not to the costal cartilage. Most of the lesion was located not in the lung, but in the mediastinal fatty connective tissue. We thoroughly observed the running of a pleural elastic layer by elastic fiber staining (Elastica van Gieson). The elastic layer of the visceral pleura bent in the way to be convex in the lung near the marginal part of the tumor, and its running manner became intermittent as it reached toward the center of the tumor, which finally disappeared. We could consider this may indicate that the primary anterior mediastinum tumor invaded into the lung. The final pathologic diagnosis was thymic LCNEC, Masaoka stage III, and T3N0M stage IIIA. Five months after surgery, CT showed pleural dissemination and left lung metastasis. The patient was given palliative care and died of the original disease 12 months after surgery.

## Discussion

Thymic LCNEC is a high-grade thymic tumor composed of large cells with neuroendocrine morphology and either neurosecretory granules on electron microscopy or positive neuroendocrine immunohistochemical markers [3]. Thymic LCNECs are very rare, accounting for only approximately 2 to 4% of all anterior mediastinal neoplasms [4]. Males are affected twice as often as females, and the median patient’s age is 51 years (ranging from 16 to 79 years) [3]. About 75% of tumors are in an advanced stage, with the invasion to neighboring organs or distant metastases (e.g., to the spine and liver) [3].

Among all the past reports of resected thymic LCNECs (Table 1) [5–21], the present patient was the oldest. Operability may often be discussed when considering surgery for elderly patients. This patient also had a co-existing illness, like other old patients, namely chronic renal failure. Though he was the oldest patient, he had good performance status preoperatively. His physical condition seemed sufficient to tolerate surgery. In fact, he had no postoperative morbidity. He was thought to be fit for surgery in the short-term perspective. As in past reports, however, he developed early recurrence. Because the natural course of non-surgical cases of thymic LCNEC is unclear, it was hard to anticipate his remaining years if surgery had not been performed. It was very hard to give him intense chemotherapies due to his poor kidney function. The only curative intent treatment was resection, because of the lack of proved effective chemotherapy, radiotherapy, or chemoradiotherapy.

**Table 1:** Reports about resected thymic large cell neuroendocrine carcinoma

Year	Author	Age (y)/sex	CT size (mm)	Preoperative therapy	Procedure	pMasaoka stage	Postoperative therapy	Relapse	Relapse date (month)	Relapse site	Observation period (months)	Outcome
2006	Nagata	57/F	70	N.A	TTH	IIB	CMT	Yes	7	Lung	11	Alive
2008	Mega	67/F	50	N.A	TTH	IVb	CMRT	Yes	6	Brain, bone	9	Dead
2009	Dutta	44/M	80	N.A	TR	N.A	CMRT	Yes	7	Lumbar vertebrae	13	N.A
2010	Cardillo	48/M	N.A	CT	THTH	III	RT	No	–	–	73	Alive
2010	Cardillo	49/M	N.A	CRT	THTH	III	RT	No	–	–	69	Alive
2010	Cardillo	50/F	N.A	N.A	THTH	IVa	RT	No	–	–	51	Dead
2010	Cardillo	48/F	N.A	N.A	THTH	III	RT	No	–	–	13	Alive
2010	Cardillo	46/M	N.A	N.A	THTH	III	RT	No	–	–	95	Dead
2010	Ogawa	55/M	42	N.A	ETH	N.A	CMT	No	–	–	16	Alive
2011	Adachi	65/F	55	N.A	TTH	IVb	None	Yes	8	Cervical LN, pleura	34	Dead
2011	Ogawa	59/F	59	N.A	ETH	III	RT	No	–	–	6	Alive
2011	Saito	38/M	60	N.A	TR	N.A	CMRT	Yes	7	Axillary LN, lung	7	Alive
2012	Machino	60/F	13	N.A	PTH	I	None	No	–	–	24	Alive
2012	Yoon	64/M	N.A	N.A	TR	IVb	None	Yes	18	Liver, adrenal gland, bone	48	Alive
2012	Yoon	57/M	N.A	CT	TR	IVb	RT	Yes	N.A	Chest wall, bone	12	Alive
2012	Ahn	67/M	N.A	RT	THTH	IVb	CMRT	Yes	3	Local	3	N.A
2012	Ahn	42/M	N.A	CRT	THTH	III	None	Yes	1	Spine	7	N.A
2012	Ahn	72/F	N.A	CT	THTH	Iva	RT	Yes	2	Mediastinum, bone, liver	4	N.A
2013	Ose	44	10	CT	TR	N.A	None	No	–	–	36	Alive
2014	Takemoto	71	60	N.A	TR	N.A	None	Yes	12	LN, liver, bone	19	Alive
2014	Yasumoto	55	52	N.A	TTH	IVb	CMT	No	–	–	10	Alive
2015	Nisizawa	68	65	N.A	PTH	II	CMT	No	–	–	17	Alive
2015	Kaiho	51	72	CT	TR	N.A	RT	No	–	–	15	Alive
2016	Kuroda	75	36	N.A	PTH	I	None	No	–	–	57	Alive
2018	Our case	90	31	None	PTH	III	None	Yes	5	Lung, pleura	12	Dead

*N.A* not available, *CMRT* chemoradiotherapy, *CT* chemotherapy, *RT* radiotherapy, *CRT* chemoradiation, *MS* median sternotomy, *VATS* video-assisted thoracic surgery, *HC* hemiclamshell, *TTH* total thymectomy, *TR* tumorectomy, *THTH* thymothymectomy, *ETH* extended thymectomy, *PTH* partial thymectomy, *LN* lymph node

It has been reported that thymic LCNEC is a heterogeneously enhanced tumor on chest CT [9, 12, 17, 19–21]. There have been only a few reports about the MRI and FDG-PET findings of thymic LCNECs. Reported MRI findings included iso- to high intensity on T1-weighted imaging and low-intensity on T2-weighted imaging [10, 13, 19]. The SUVmax of thymic LCNEC was reported to be very high, 13.5 [17], 17.1 [21], and 20.7 [20]. In the present case, CT was performed without contrast media because of the patient’s chronic kidney failure. Plain CT showed a homogeneous mass with a low-density septal wall. Slightly different from the previous report, MRI, in this case, showed iso-intensity of the mass on T1-weighted images and slightly high intensity on T2-weighted images. FDG-PET showed a high SUVmax, as in the previous report. Considering the very high SUVmax, this tumor was thought to have high malignant potential, likely representing thymic carcinoma or invasive thymoma. However, thymic LCNEC was not suspected preoperatively because of its rarity. In any case, if high malignant potential is suspected, total thymectomy via median sternotomy would usually be preferred. Because the patient was super-elderly (90 years old) with some comorbidities, however, we selected the operation method with the least invasiveness, VATS partial thymectomy.

When examining tumor presenting in both the mediastinum and lung, it is extremely important to determine whether the lesion represents mediastinal tumor or primary lung cancer with mediastinal invasion. We sometimes encounter cases of lung cancer with a large volume of mediastinal invasion compared with the pulmonary part. We think that the origin in this case was the mediastinum because of the lesion site. Almost all of the tumors were present in the mediastinum, not in the lung. Histopathological examination showed the volume of tumor invading the lung was quite small. Tumor volume in the lung was too small to consider the lung as the primary organ. On the other hand, several reports [22–24] have described immunohistochemical examination of thymic carcinoma showing positive results for CD5, c-kit, or PAX8. However, most of those reports involved thymic squamous cell carcinoma. No articles appear to have described methods of immunohistochemical discrimination for thymic LCNEC. In the present case, this problem was impossible to solve immunohistochemically.

Thymic LCNEC seems to have distant metastases even in the early stage. Reported 5-year overall survival ranges from 30 to 66% [3]. Cardillo et al. reported that the 10-year survival rate was 0% in thymic LCNEC [25]. The Japanese guideline for thymic epithelial tumors recommends postoperative radiotherapy following complete resection of thymic carcinomas. Tiffet et al. reported a patient who survived for 67 months without recurrence after complete resection of thymic LCNEC followed by adjuvant radiotherapy [26]. The guideline does not mention the effectiveness of chemotherapy after complete resection of thymic epithelial cancer. Although there is no evidence to support adjuvant therapy for thymic LCNEC, a regimen using cisplatin/carboplatin/etoposide, as for lung small cell carcinoma, seems the most common choice in thymic LCNEC at this time [21]. Nagata et al. reported a case of lung metastasis of thymic LCNEC after four courses of carboplatin/etoposide that achieved complete response [5]. Although such responders were sometimes seen, these all have a major limitation due to the small numbers of patients. Further consideration of radiotherapy and chemotherapy in thymic LCNEC is needed.

## Conclusion

Thymic LCNEC has high malignant potential. Though resection is currently the optimal first treatment option, the prognosis remains poor. To clarify the best treatment for thymic LCNEC, many cases of thymic LCNEC need to be studied.

## Abbreviations

CMRT: Chemoradiotherapy
CMT: Chemotherapy
CT: Computed tomography
ETH: Extended thymectomy
FDG-PET: 18F-Fluorodeoxyglucose-positron emission tomography
HC: Hemiclamshell
LCNEC: Large cell neuroendocrine carcinoma
LN: Lymph node
MRI: Magnetic resonance imaging
MS: Median sternotomy
N.A: Not available
NEC: Neuroendocrine carcinoma
PTH: Partial thymectomy
RT: Radiotherapy
SUVmax: Maximum standardized uptake value
THTH: Thymothymectomy
TR: Tumorectomy
TTF-1: Thyroid transcription factor-1
TTH: Total thymectomy
VATS: Video-assisted thoracic surgery
